# Case Report: Giant adult rhabdomyoma of the parapharyngeal space: diagnostic and surgical pitfalls

**DOI:** 10.3389/fmed.2026.1945205

**Published:** 2026-09-25

**Authors:** Bingyi Dai, Hongyang Qu, Rong Liu, Changling Sun, Xiao Yin

**Affiliations:** Department of Otolaryngology–Head and Neck Surgery, Affiliated Hospital of Jiangnan University, Wuxi, China

**Keywords:** adult rhabdomyoma, case report, differential diagnosis, dysphagia, immunohistochemistry, parapharyngeal space, transcervical approach

## Abstract

**Background:**

Adult-type extracardiac rhabdomyoma is a rare benign skeletal-muscle neoplasm that most often arises in the head and neck but is exceptional in the parapharyngeal space, where it may mimic salivary or neurogenic tumours. Case presentation: A 67-year-old man presented with a 6-month history of progressive dysphagia, globus sensation, and mild hoarseness. Computed tomography and magnetic resonance imaging demonstrated an approximately 9-cm, well-circumscribed right parapharyngeal mass with relatively homogeneous internal characteristics and mild-to-moderate enhancement. Because direct glottic exposure was inadequate, tracheotomy-assisted airway management preceded complete excision through a right lateral transcervical approach. Histopathology showed large polygonal cells with abundant eosinophilic granular cytoplasm. The tumour was positive for desmin and smooth-muscle actin, with focal MyoD1 and myogenin expression, a low Ki-67 labelling index, and negative S-100 staining, supporting adult-type rhabdomyoma. Recovery was uneventful, and magnetic resonance imaging at 6 and 12 months showed no recurrence.

**Conclusion:**

Adult rhabdomyoma should be considered in the differential diagnosis of a well-circumscribed parapharyngeal mass in an older adult. Integrated radiological and pathological assessment, careful airway planning, complete conservative excision, and long-term surveillance are central to management.

## Introduction

Adult-type extracardiac rhabdomyoma is a rare benign neoplasm showing mature skeletal-muscle differentiation (1–3). Extracardiac rhabdomyomas are histologically classified into adult, fetal and genital types, and the adult type has a strong predilection for the head and neck, particularly the pharynx, larynx, oral cavity and soft palate (1, 4–7). Even within this anatomical distribution, origin in the parapharyngeal space is exceptional, with published evidence largely restricted to isolated reports and small series (4–7). Its rarity, deep location and nonspecific symptoms create a substantial risk of preoperative misclassification as a salivary, neurogenic or paraganglionic tumour.

The parapharyngeal space contains major vessels, lower cranial nerves, sympathetic structures, fat and deep-lobe salivary tissue. Tumours arising in this compartment account for only a small proportion of head and neck neoplasms and are predominantly benign; pleomorphic adenomas and neurogenic tumours represent the most frequent pathological groups (8–11). Clinical manifestations are determined mainly by tumour size and direction of extension. Slowly enlarging lesions may therefore remain clinically occult until they cause dysphagia, globus sensation, voice change, snoring, otologic symptoms or cranial-nerve dysfunction (12).

Cross-sectional imaging is indispensable for localizing a parapharyngeal lesion, defining its relationship to the carotid sheath and skull base, and selecting a safe surgical corridor (9–11, 13). However, the imaging phenotype of adult rhabdomyoma overlaps with that of several more common benign tumours (4, 6, 7, 13), and definitive diagnosis generally depends on integrated morphology, immunohistochemistry and, when obtained, cytological assessment (1, 6, 14, 15). We report a giant adult-type rhabdomyoma of the right parapharyngeal space in a 67-year-old man and provide a focused review of the diagnostic traps, pathological differential diagnosis, approach selection and recurrence considerations relevant to this rare entity.

## Case presentation

### Patient information and clinical findings

A 67-year-old man presented after 6 months of progressive dysphagia and globus sensation, which were his principal concerns. He also reported mild odynophagia and hoarseness. He denied fever, weight loss, night sweats and other constitutional symptoms.

The de-identified record contained no documented medical or surgical comorbidity relevant to the present illness, regular medication or drug allergy relevant to perioperative care. He had not undergone biopsy, surgery or other treatment for the lesion, so there was no earlier intervention outcome to report. He reported no known family history of tumour predisposition or recognised hereditary syndrome; genetic testing was not performed. No psychosocial factor affecting assessment, treatment or follow-up was documented.

Examination showed mild smooth bulging of the right lateral pharyngeal wall without an obvious external cervical mass or documented cranial-nerve deficit. The pharyngeal mass narrowed the upper airway, making difficult-airway planning a key perioperative concern.

## Clinical timeline

The patient's presentation, diagnostic assessment, treatment, and follow-up are summarized in Table 1.

**Table 1:** CARE-aligned timeline of presentation, diagnostic assessment, treatment and follow-up.

Time point	Clinical event	Key findings, decision or outcome
Approximately 6 months before presentation	Symptom onset	Progressive dysphagia and globus sensation, followed by mild odynophagia and hoarseness
Preoperative clinical assessment	History, head-and-neck examination and airway assessment	Smooth right lateral pharyngeal-wall bulge, no external cervical mass or documented cranial-nerve deficit, and clinically important upper-airway narrowing
Preoperative imaging	CT and contrast-enhanced MRI	Approximately 9-cm, well-circumscribed right parapharyngeal mass with relatively homogeneous internal characteristics, mild-to-moderate enhancement, airway compression and no destructive change
15 August 2024	Airway management	Direct glottic exposure was inadequate; the airway plan was changed to tracheotomy-assisted endotracheal intubation before general anaesthesia
15 August 2024	Definitive treatment	Intact excision through a right lateral transcervical approach with identification and preservation of the major cervical neurovascular structures
Postoperative pathological assessment	Histology and immunohistochemistry	Adult-type rhabdomyoma supported by characteristic morphology, skeletal-muscle-marker expression, low Ki-67 labelling and negative S-100 staining
Early postoperative period	Clinical recovery	No major haemorrhage, airway or wound complication, or new lower cranial-nerve deficit; dysphagia and hoarseness improved
6 months after surgery	Clinical and MRI surveillance	No residual or recurrent tumour
12 months after surgery	Clinical and MRI surveillance	No evidence of recurrence; continued long-term follow-up advised

### Diagnostic assessment and differential diagnosis

Computed tomography demonstrated a well-demarcated, relatively homogeneous soft-tissue mass in the right parapharyngeal space, without obvious calcification, haemorrhage, necrosis or adjacent bone destruction. Magnetic resonance imaging showed a large, sharply circumscribed lesion measuring approximately 9 cm in maximum dimension. It was low to intermediate in signal intensity on T1-weighted images, high in signal intensity on T2-weighted images and mildly to moderately and relatively homogeneously enhanced after contrast administration. The mass compressed the pharyngeal airway but remained clearly delineated from adjacent structures on the available images (Figures 1a–f).

**Figure 1:**
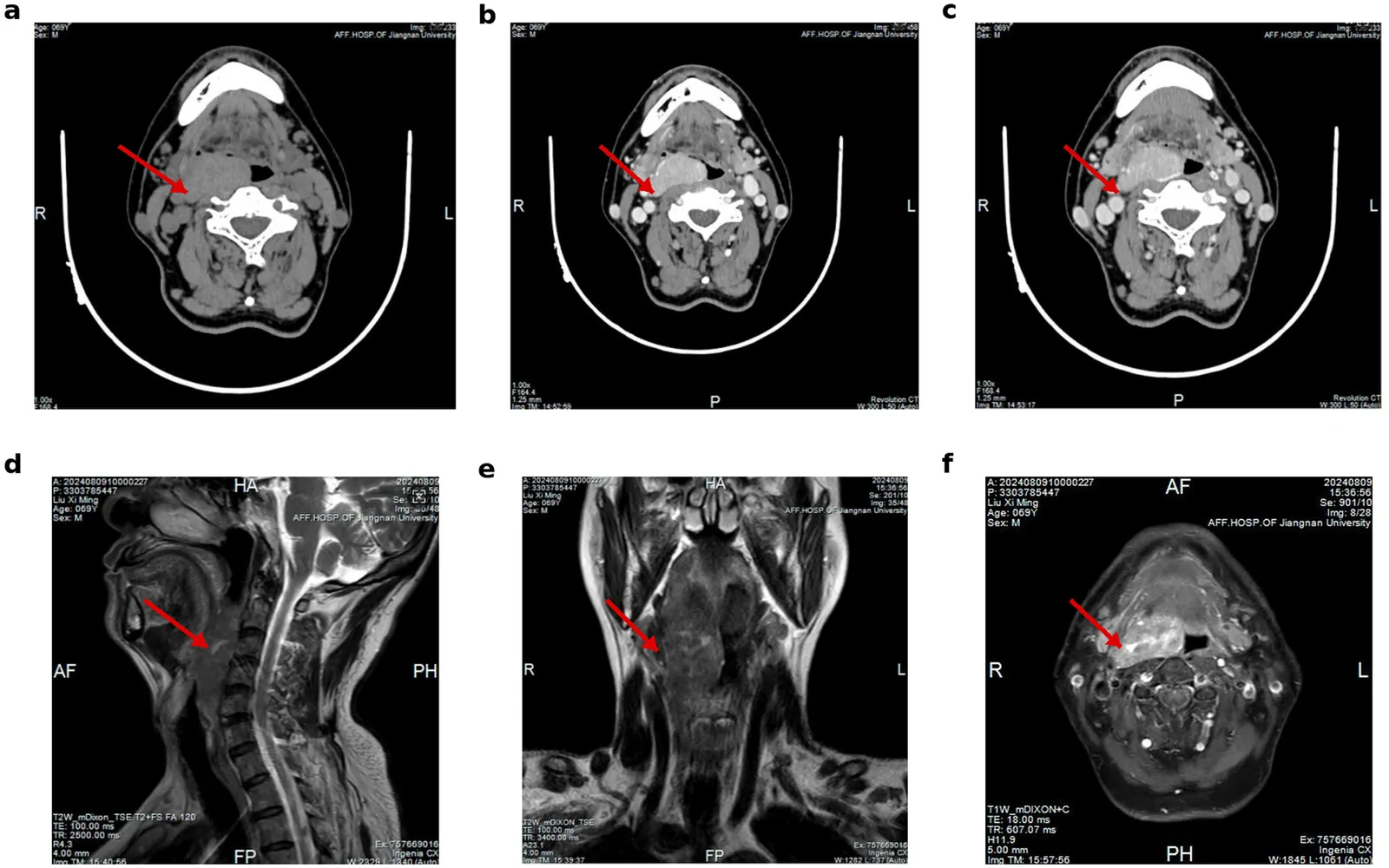
Preoperative imaging of the right parapharyngeal-space tumour. **(a–c)** Axial computed tomography images show a well-circumscribed right parapharyngeal soft-tissue mass. **(d,e)** Sagittal and coronal magnetic resonance images delineate its craniocaudal extent and relationship to the pharyngeal airway. **(f)** Axial contrast-enhanced magnetic resonance image shows mild-to-moderate enhancement. Red arrows indicate the tumour.

The circumscribed margins, relative homogeneity and absence of destructive change favoured a benign, slowly growing tumour but were not diagnostic. The preoperative differential diagnosis included a pleomorphic adenoma arising from deep-lobe or minor salivary tissue, schwannoma, neurofibroma, paraganglioma and other mesenchymal tumours. Adult-type rhabdomyoma was not the leading preoperative diagnosis, illustrating how readily this lesion can be overlooked when it arises in an unusual deep-neck compartment.

### Therapeutic intervention and operative findings

Complete conservative excision was recommended because the approximately 9-cm lesion caused progressive swallowing and voice symptoms and compressed the upper airway. A right lateral transcervical route was selected to provide direct access and reliable control of the carotid sheath and lower cranial nerves. Surgery was performed on 15 August 2024. When the glottis could not be adequately exposed for intubation, the airway plan was changed to tracheotomy-assisted endotracheal intubation before general anaesthesia; this change secured the airway before deep-neck dissection.

The patient was positioned supine with the head turned to the left. An approximately 5-cm transverse cervical incision was made, subplatysmal flaps were elevated and the lesion was approached medial to the upper sternocleidomastoid muscle. The tumour was smooth, dark purple and only mildly adherent to adjacent tissues; needle aspiration did not yield blood. The internal jugular vein, common carotid artery and vagus nerve were identified and protected. Dissection proceeded superiorly along the prevertebral plane toward the skull base and the superior border of the digastric muscle.

The mass was removed intact and measured approximately 9 × 4 × 2 cm. Facial-vein bleeding was repaired with 7-0 Prolene without altering the intended conservative resection. After meticulous haemostasis, a negative-pressure drain was placed and the wound was closed in layers. No wider oncological resection or adjuvant tumour-directed therapy was indicated after complete excision and confirmation of a benign adult-type rhabdomyoma.

The postoperative course was uneventful. Clinician assessment documented no major haemorrhage, airway compromise, wound complication or new lower cranial-nerve deficit, while the patient reported gradual improvement in dysphagia and hoarseness. He attended clinical and MRI surveillance at 6 and 12 months; both examinations showed no residual or recurrent tumour. Because treatment consisted of a one-time operation and no adjuvant tumour-directed therapy was prescribed, ongoing treatment adherence did not apply. No treatment-limiting adverse or unanticipated event was documented. Continued long-term surveillance was recommended because local recurrence is reported particularly after incomplete excision and in multifocal disease (16–18).

### Histopathological and immunohistochemical diagnosis

Gross examination showed a well-demarcated, lobulated, red-brown to grey-white mass with a smooth surface and a relatively homogeneous cut surface (Figure 2a). Microscopically, the tumour consisted of large round-to-polygonal cells with distinct borders, abundant eosinophilic granular cytoplasm and eccentric round-to-oval nuclei (Figure 2b). Peripheral vacuolation compatible with glycogen-rich cytoplasm was present in representative areas. No overt cytological atypia, tumour necrosis or brisk mitotic activity was identified in the submitted material.

**Figure 2:**
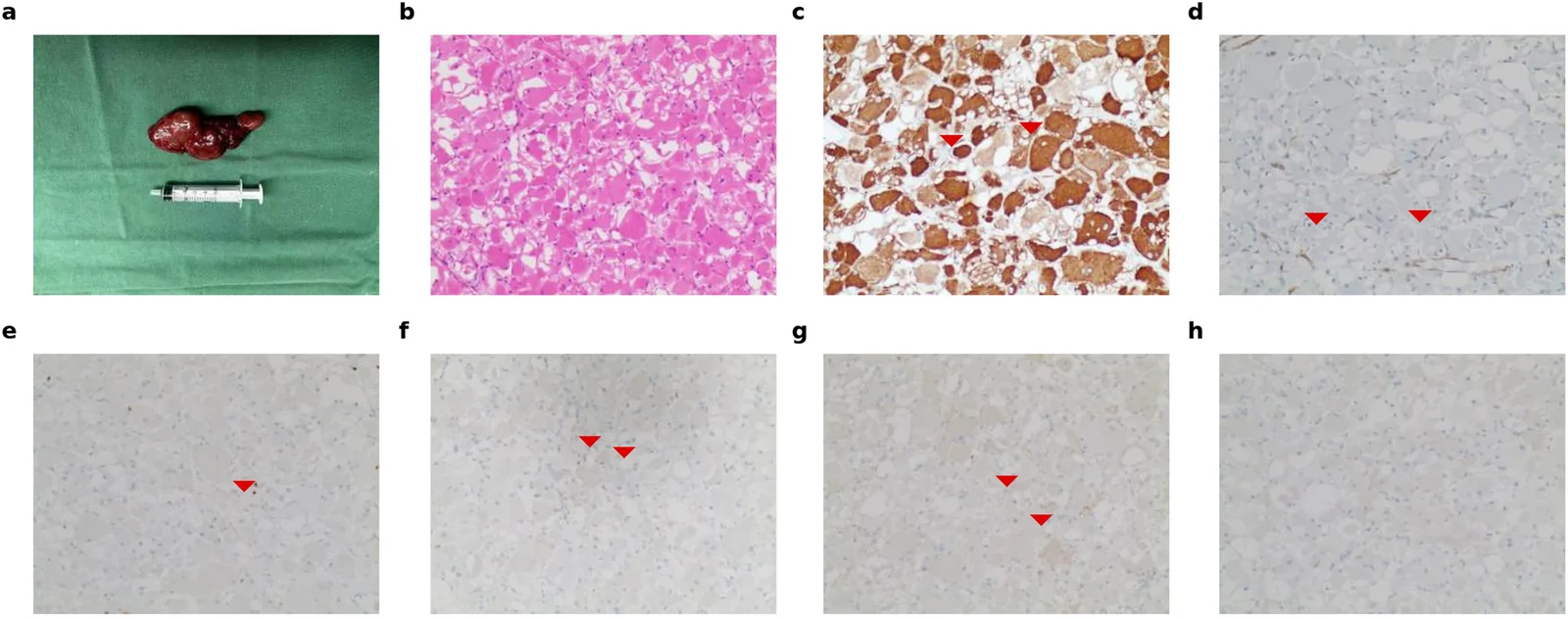
Gross, histopathological and immunohistochemical findings. **(a)** Gross specimen showing a lobulated, red-brown, well-demarcated mass. **(b)** Haematoxylin and eosin staining showing large polygonal cells with abundant eosinophilic granular cytoplasm and eccentric nuclei. **(c)** Desmin positivity. **(d)** Smooth-muscle-actin positivity. **(e)** Low/scattered Ki-67 labelling. **(f)** Scattered MyoD1 expression. **(g)** Scattered myogenin expression. **(h)** Absence of S-100 staining in the submitted section. Red arrowheads indicate positive immunohistochemical signals; no arrowhead is shown in panel h because S-100 staining was negative.

Immunohistochemistry showed cytoplasmic positivity for desmin and smooth-muscle actin, low/scattered Ki-67 labelling, scattered MyoD1 and myogenin expression, and no S-100 expression in the submitted sections (Figures 2c–h). Positive signals are marked by red arrowheads in the relevant panels. In combination with the characteristic morphology and indolent behaviour, the immunophenotype supported adult-type rhabdomyoma. The principal pathological differentials were granular cell tumour, paraganglioma, hibernoma, neurogenic tumour and well-differentiated rhabdomyosarcoma.

## Methods: focused literature review

This article combines a single-patient case report with a focused narrative literature review. Clinical presentation, imaging, operative findings, histopathology, immunohistochemistry and follow-up were extracted from the patient's medical record and the authors' source material. No inferential statistical analysis was performed. The case narrative was organized with reference to the CARE reporting framework.

PubMed was searched through 31 August 2026, supplemented by screening the reference lists of relevant articles, using combinations of the terms ‘adult rhabdomyoma’, ‘extracardiac rhabdomyoma’, ‘parapharyngeal space’, ‘head and neck’, ‘imaging’, ‘differential diagnosis’, ‘granular cell tumour’ and 'surgical approach'. Reports, case series and reviews were used to contextualize clinical presentation, radiological and pathological differential diagnosis, operative-corridor selection, conservative management and recurrence. Because the purpose was focused clinical synthesis rather than exhaustive evidence aggregation, the review was not registered, and no meta-analysis was performed.

## Discussion

Adult rhabdomyoma is histologically benign, slow growing and non-metastatic, yet a parapharyngeal lesion can become clinically consequential because of progressive compression of the pharyngeal airway, swallowing pathway, eustachian tube, carotid sheath or lower cranial nerves. Published experience remains dominated by isolated cases and small series (4–7, 16). Across these reports, presentation is typically delayed and driven by mass effect rather than tumour-specific symptoms. The progressive dysphagia, globus sensation and mild hoarseness in our patient were therefore anatomically coherent but diagnostically nonspecific.

The first major pitfall is radiological overclassification. CT establishes density, calcification, osseous change and vascular displacement, whereas MRI more accurately defines soft-tissue planes, carotid-sheath relationships and skull-base extension (8–11, 13). Adult rhabdomyoma is often well circumscribed and may enhance relatively uniformly, but these features overlap with pleomorphic adenoma and schwannoma. Paraganglioma is more likely to show marked vascular enhancement and flow-related signal voids, whereas an infiltrative margin, necrosis, nodal disease or destructive change should raise concern for malignancy. In the present case, imaging correctly suggested a benign process and guided operative planning, but it could not reliably establish the histological diagnosis.

The second pitfall is pathological mimicry. Adult rhabdomyoma classically contains large polygonal cells with abundant eosinophilic granular cytoplasm, glycogen-related vacuolation and, occasionally, visible cross-striations. Desmin, myoglobin, MyoD1 and myogenin support skeletal-muscle differentiation, although the extent of staining may vary (1, 3, 6, 14, 15). Granular cell tumour may closely resemble rhabdomyoma morphologically but is of Schwann-cell lineage, usually shows diffuse S-100/SOX10 expression and lacks convincing skeletal-muscle differentiation (19, 20). Paraganglioma has a nested neuroendocrine architecture with chromogranin and synaptophysin expression in chief cells. Hibernoma shows multivacuolated brown-fat differentiation, while rhabdomyosarcoma is distinguished by destructive growth, cytological atypia, mitotic activity and a substantially higher proliferative fraction. Importantly, S-100 positivity has occasionally been reported in rhabdomyoma (21); the diagnosis should therefore rest on integrated morphology and a marker panel rather than any single stain.

Management should be proportionate to symptoms, growth and anatomical risk. Observation has been proposed for small asymptomatic lesions and selected multifocal disease (17, 22), whereas complete conservative excision remains the preferred treatment for a symptomatic or enlarging adult rhabdomyoma. Wide oncological margins and adjuvant therapy are not indicated after complete excision of a benign lesion. Surgical approach selection should be individualized according to prestyloid or poststyloid location, tumour size, carotid relationship, skull-base extension, airway effects and the surgeon's ability to control haemorrhage (8–11). A transcervical route provides familiar exposure and vascular control for large or poststyloid lesions. Endoscope-assisted or robot-assisted transoral surgery can reduce external morbidity in carefully selected encapsulated, prestyloid and carotid-medial tumours, but limited working space and haemorrhage control remain important constraints (23–25). Endonasal transpterygoid and lateral skull-base corridors are reserved for selected high parapharyngeal or skull-base lesions and require a morbidity-conscious indication (26–29). Table 2 summarizes these approach-selection principles.

**Table 2:** Surgical approach selection for parapharyngeal-space tumours relevant to adult rhabdomyoma.

Approach	Typical indication	Main advantage	Main limitation
Transcervical	Large middle or lower parapharyngeal-space lesions; poststyloid lesions; close relationship to major vessels	Wide exposure and vascular control; familiar route for many head-and-neck surgeons	External scar; limited superior/skull-base exposure in selected cases
Transcervical-parotid	Tumours involving the deep parotid lobe or requiring facial-nerve identification	Improved access to prestyloid/deep-lobe lesions and high parapharyngeal tumours	Facial-nerve morbidity, Frey syndrome and parotid contour change
Endoscope-assisted transoral	Selected benign, encapsulated, prestyloid, carotid-medial tumours with limited skull-base extension	No external scar; short recovery; improved medial and superior visualization with endoscopic assistance	Restricted working space; bleeding control and tumour rupture risk; not ideal for poststyloid or very large lesions
Endoscopic transnasal transpterygoid	Selected upper parapharyngeal or infratemporal extension close to the skull base	Direct endoscopic access to high lesions without mandibulotomy	Technically demanding; narrow corridor; limited role for large lateral or vascular tumours
Mandibulotomy or lateral skull-base approaches	Very large, recurrent, malignant or skull-base-extending tumours requiring maximal exposure	Excellent exposure and control of high or complex disease	Greater morbidity, longer recovery and risk of mandibular, dental, sensory or functional complications

The literature and the present operative experience converge on three practical lessons. First, adult rhabdomyoma should enter the differential diagnosis of a well-circumscribed, slowly enlarging parapharyngeal mass in an older adult, even when imaging initially favours a salivary or neurogenic tumour. Second, diagnosis requires clinicoradiological-pathological integration because both imaging and isolated immunostains can mislead. Third, treatment must be individualized: the goal for a large symptomatic lesion is intact conservative excision with preservation of neurovascular function, whereas observation may be reasonable in selected asymptomatic patients. The distinctive contribution of this case is the combined documentation of a giant lesion, difficult airway management, transcervical neurovascular dissection, immunophenotypic confirmation and short-term radiological follow-up. Computational modelling of tumour-related tracheal compression further supports the importance of anticipating airflow compromise during airway planning (30).

The strengths of this report are the concordant CT and MRI assessment, explicit documentation of the airway and operative decisions, examination of an intact specimen by morphology and an immunohistochemical panel, patient- and clinician-assessed postoperative outcomes, serial MRI surveillance, and a CARE-aligned timeline.

Several limitations temper interpretation. This is a retrospective single-patient report and cannot estimate incidence or recurrence risk, establish causality, or compare surgical approaches. Some medical, psychosocial and genetic-history elements were not documented in the de-identified source record; no preoperative tissue diagnosis was obtained; and routine imaging and pathological material were not acquired under a prospective standardized protocol. The focused review was narrative, relied primarily on PubMed and reference screening, and is therefore susceptible to publication and selection bias. Follow-up of 12 months cannot exclude late recurrence, and no standardized patient-reported outcome measure or longer patient-authored narrative was available. Accordingly, the findings support diagnostic awareness and management principles for anatomically similar cases but should not be generalized as evidence that one operative route is superior.

## Conclusion

Adult rhabdomyoma should be considered when an older adult has a slowly enlarging, well-circumscribed parapharyngeal mass, even if imaging favours a salivary or neurogenic tumour. Diagnosis depends on integration of imaging, morphology and immunohistochemistry. For a large symptomatic lesion, careful airway planning and complete conservative excision can relieve compression while preserving neurovascular function; continued surveillance is prudent because multifocality and late local recurrence may be overlooked.

## Patient perspective

At follow-up, the patient reported that swallowing and voice had improved after surgery. A longer patient-authored account was not available for inclusion.

## Data Availability

The original contributions presented in this case report are included in the article; further inquiries can be directed to the corresponding author.

## References

[1] Kapadia SB Meis JM Frisman DM Ellis GL Heffner DK Hyams VJ. Adult rhabdomyoma of the head and neck: a clinicopathologic and immunophenotypic study. Hum Pathol. (1993) 24:608–17. 10.1016/0046-8177(93)90240-H 8505039

[2] Di Sant'Agnese PA Knowles DM 2nd. Extracardiac rhabdomyoma: a clinicopathologic study and review of the literature. Cancer. (1980) 46:780–9. 10.1002/1097-0142(19800815)46:4<780::AID-CNCR2820460423>3.0.CO;2-K 10.1002/1097-0142(19800815)46:4<780::aid-cncr2820460423>3.0.co;2-k 7397640

[3] Willis J Abdul-Karim FW di Sant'Agnese PA. Extracardiac rhabdomyomas. Semin Diagn Pathol. (1994) 11:15–25. 8202643

[4] Papaspyrou G Werner JA Roeßler M Devaney KO Rinaldo A Ferlito A. Adult rhabdomyoma in the parapharyngeal space: report of 2 cases and review of the literature. Am J Otolaryngol. (2011) 32:240–6. 10.1016/j.amjoto.2010.01.007 20392534

[5] Pichi B Manciocco V Marchesi P Pellini R Ruscito P Vidiri A. Rhabdomyoma of the parapharyngeal space presenting with dysphagia. Dysphagia. (2008) 23:202–4. 10.1007/s00455-007-9125-0 17965913

[6] Bjørndal Sørensen K Godballe C Ostergaard B Krogdahl A. Adult extracardiac rhabdomyoma: light and immunohistochemical studies of two cases in the parapharyngeal space. Head Neck. (2006) 28:275–9. 10.1002/hed.20358 16419079

[7] Gatsounia A Bosgana P Danielides G Vlachodimitropoulos A Kurabi H Lygeros S. A rare case of adult rhabdomyoma in the parapharyngeal space: diagnostic challenges and surgical management. Case Rep Pathol. (2025) 2025:1626816. 10.1155/crip/1626816 41210894 PMC12595224

[8] Riffat F Dwivedi RC Palme C Fish B Jani P. A systematic review of 1143 parapharyngeal space tumors reported over 20 years. Oral Oncol. (2014) 50:421–30. 10.1016/j.oraloncology.2014.02.007 24589290

[9] López F Suárez C Vander Poorten V Mäkitie A Nixon IJ Strojan Pž. Contemporary management of primary parapharyngeal space tumors. Head Neck. (2019) 41:522–35. 10.1002/hed.25439 30549361

[10] Strohl MP El-Sayed IH. Contemporary management of parapharyngeal tumors. Curr Oncol Rep. (2019) 21:103. 10.1007/s11912-019-0853-8 31728649

[11] Jiang C Wang W Chen S Liu Y. Management of parapharyngeal space tumors: clinical experience and treatment strategy. Curr Oncol. (2023) 30:593–605. 10.3390/curroncol30010078 PMC9857702 36661727

[12] Gilli M Committeri U Monarchi G Bimonte A Caso AR Sacchi F. Giant lipoma of the neck causing airways compression: a case report. Oral Surg. (2026) 19:132–7. 10.1111/ors.70041

[13] Helmberger RC Stringer SP Mancuso AA. Rhabdomyoma of the pharyngeal musculature extending into the prestyloid parapharyngeal space. AJNR Am J Neuroradiol. (1996) 17:1115–8. 8791925 PMC8338621

[14] Dermawan JK Doxtader E Chute DJ Policarpio-Nicolas ML. Cytologic findings of an adult rhabdomyoma in the parapharyngeal space: a report of a case and review of the literature. Diagn Cytopathol. (2018) 46:419–24. 10.1002/dc.23860 29131558

[15] Zhang S Bhalodia A Swartz B Abreo F Fowler M. Fine needle aspiration of parapharyngeal space adult rhabdomyoma: a case report. Acta Cytol. (2010) 54:775–9. 21053538

[16] Gajda M Welzel C Holzhausen HJ Jamali Y Schrom T Hauptmann S. Multifocal adult rhabdomyoma of the neck: a rare entity. Otolaryngol Pol. (2005) 59:883–6. 16521457

[17] Khalaf MG Haddad R Akiki M Khazen J Melkane AE. Multifocal adult rhabdomyoma of the head and neck: case report and systematic review of the literature. Int J Oral Maxillofac Surg. (2021) 50:327–34. 10.1016/j.ijom.2020.07.018 32773112

[18] Jotić A Jakovljević S Tomanović N Jovanović K Glogovac H. The return of a rare entity: recurrent neck rhabdomyoma. J Craniofac Surg. (2026). 10.1097/SCS.0000000000013291 42598796

[19] Ardeleanu V Jecan RC Moroianu M Teodoreanu RN Tebeica T Moroianu LA. Case report: abrikossoff’s tumor of the facial skin. Front Med. (2023) 10:1149735. 10.3389/fmed.2023.1149735 PMC10264634 37324160

[20] Hou J Zheng Y. A rare case of intramuscular granular cell tumor in the right thigh: case report and literature review. Front Med. (2026) 13:1822058. 10.3389/fmed.2026.1822058 PMC13337469 42415803

[21] Palicelli A Ramponi A Valente G Boldorini R Mussetto BA Zanelli M. S-100 immunohistochemical positivity in rhabdomyoma: an underestimated potential diagnostic pitfall in routine practice. Diagnostics. (2022) 12:892. 10.3390/diagnostics12040892 35453940 PMC9030831

[22] Khan AM Chor PJ Eisenbeis JF. Adult extracardiac rhabdomyoma of the parapharyngeal space. Ear Nose Throat J. (2012) 91:E7–10. 22996720

[23] Larson AR Ryan WR. Transoral excision of parapharyngeal space tumors. Otolaryngol Clin North Am. (2021) 54:531–41. 10.1016/j.otc.2021.03.001 34024481

[24] Lao WP Han PS Lee NH Gilde JE Inman JC. Transoral excision of parapharyngeal tumors. Ear Nose Throat J. (2021) 100:NP454–8. 10.1177/0145561320923171 32425121

[25] Brady JS Miller C Ahuja S Rogers DM Rizvi ZH. Transoral robotic surgical resection of bilateral parapharyngeal space rhabdomyoma. Case Rep Otolaryngol. (2025) 2025:2786874. 10.1155/crot/2786874 41473204 PMC12747048

[26] Li L London N Prevedello DM Carrau RL. Endonasal endoscopic transpterygoid approach to the upper parapharyngeal space. Head Neck. (2020) 42:2734–40. 10.1002/hed.26127 32129556

[27] Wasano K Yamamoto S Tomisato S Kawasaki T Ogawa K. Modified endoscopic transnasal-transmaxillary-transpterygoid approach to parapharyngeal space tumor resection. Head Neck. (2016) 38:933–8. 10.1002/hed.24399 26855115

[28] Battaglia P Turri-Zanoni M Dallan I Gallo S Sica E Padoan G. Endoscopic endonasal transpterygoid transmaxillary approach to infratemporal and upper parapharyngeal tumors. Otolaryngol Head Neck Surg. (2014) 150:696–702. 10.1177/0194599813520290 24457630

[29] Chu F Tagliabue M Giugliano G Calabrese L Preda L Ansarin M. From transmandibular to transoral robotic approach for parapharyngeal space tumors. Am J Otolaryngol. (2017) 38:375–9. 10.1016/j.amjoto.2017.03.004 28390805

[30] Zobaer T Sutradhar A. Modeling the effect of tumor compression on airflow dynamics in trachea using contact simulation and CFD analysis. Comput Biol Med. (2021) 135:104574. 10.1016/j.compbiomed.2021.104574 34175532

